# PERCEIVED BURDEN ON OTHERS, LONELINESS, AND SUICIDE RISK IN CAREGIVERS

**DOI:** 10.1093/geroni/igae098.1237

**Published:** 2024-12-31

**Authors:** Kimberly Van Orden

**Affiliations:** University of Rochester Medical Center, Rochester, New York, United States

## Abstract

Caregiving can foster increased purpose and mastery, but also anxiety, depression, and suicide ideation. Mechanisms whereby caregiving impacts suicide risk in dementia caregivers are not well characterized. The Interpersonal Theory of Suicide posits that stressors such as caregiving increase suicide risk by producing feelings of not belong to valued relationships/groups (low belonging, loneliness) and like one is a burden on others (perceived burden). Given that caregivers by definition are caring for others, they may be protected against perceived burden. This presentation will examine the relevance of the Interpersonal Theory of Suicide to understanding mechanisms whereby caregiving may increase suicide risk, with a particular focus on perceived burden on others. Data come from 106 caregivers who reported both significant loneliness and caregiving stress (i.e., required inclusion characteristics for studies funded by Rochester Roybal Center for Social Ties & Aging Research). Subjects completed semi-structured interviews (Columbia Suicide Severity Rating Scale) and self-report measures of social disconnection (Interpersonal Needs Questionnaire), depressive symptoms, caregiving burden, and neuropsychiatric symptoms. Results indicate that 10% reported suicide ideation (past month) and 14% reported feeling like a burden on others (past month). Caregivers who reported perceived burden were 3.5 times more likely to report suicide ideation. Findings suggest that perceived burden is as least as common as suicide ideation, supporting relevance of the Interpersonal Theory. Additional analyses will examine characteristics of those who reported perceived burden to explore potential contributors to developing perceptions of burden despite providing dementia care.

